# Impact of a Mindfulness-Based Intervention on Basic Psychological Need Satisfaction and Internalized Symptoms in Elementary School Students With Severe Learning Disabilities: Results From a Randomized Cluster Trial

**DOI:** 10.3389/fpsyg.2019.02715

**Published:** 2019-12-06

**Authors:** Catherine Malboeuf-Hurtubise, Geneviève Taylor, Geneviève A. Mageau

**Affiliations:** ^1^Department of Psychology, Bishop’s University, Sherbrooke, QC, Canada; ^2^Department of Education and Pedagogy, Université du Québec à Montréal, Montreal, QC, Canada; ^3^Department of Psychology, Université de Montréal, Montreal, QC, Canada

**Keywords:** basic psychological need satisfaction, autonomy, competence, relatedness, severe learning disabilities, mindfulness, anxiety, depression

## Abstract

**Background:**

Mindfulness is hypothesized to lead to more realistic appraisals of the three basic psychological needs, which leads people to benefit from high levels of need satisfaction or helps them make the appropriate changes to improve need satisfaction. Mindfulness-based interventions (MBIs) have also shown promise to foster greater basic psychological need satisfaction in students with learning disabilities (LDs).

**Objective:**

The goal of the present study was to evaluate the impact of a MBI on the satisfaction of the basic psychological needs and on internalized symptoms in students with severe LDs. A randomized cluster trial was implemented to compare the progression of need satisfaction, anxiety, and depression symptoms in participants pre- to post-intervention and at follow-up.

**Method:**

Elementary school students with severe LDs (*N* = 23) in two special education classrooms took part in this study and were randomly attributed to either an experimental or an active control group.

**Results:**

Mixed ANOVAs first showed that the experimental condition did not moderate change over time such that similar effects were observed in the experimental and active control groups. Looking at main effects of time on participants’ scores of autonomy, competence, and relatedness across time, we found a significant within-person effect for the competence need (*p* = 0.02). *Post hoc* analyses showed that for both groups, competence scores were significantly higher at post-intervention (*p* = 0.03) and at follow-up (*p* = 0.04), when compared to pre-intervention scores. A significant main effect was also found for anxiety levels over time (*p* = 0.008). *Post hoc* analyses showed that for both groups, scores were significantly lower at post-intervention (*p* = 0.01) and at follow-up (*p* = 0.006), when compared to pre-intervention scores.

**Conclusion:**

Although the MBI seemed useful in increasing the basic psychological need of competence and decreasing anxiety symptoms in students with severe LDs, it was not more useful than the active control intervention that was used in this project. Future studies should verify that MBIs have an added value compared to other types of interventions that can be more easily implemented in school-based settings.

## Introduction

Students with learning disabilities (LDs) represent 13% of the total student population in the United States (Cortiella and Horowitz, 2014). Research has shown that teachers tend to be more controlling with students who have severe LDs than with students with no identified disability, resulting in lower perceived competence and autonomy (Grolnick and Ryan, 1990). Yet, feeling autonomous, competent, and related to others is just as important for these students as for students in regular education curriculum; high satisfaction of these needs is indeed associated with academic achievement and psychological adjustment of students with identified LDs (Deci et al., 1992; Carter et al., 2006; Konrad et al., 2007; Anctil et al., 2008). Providing opportunities for self-determined skills instruction and learning has been suggested as a cornerstone of specialized education for students with severe LDs (Field et al., 2003). Indeed, the positive effects of self-determined education can be seen later in life in students with severe LDs, as those with higher basic psychological need satisfaction secure higher-paying and more stable jobs (Wehmeyer and Schwartz, 1997). However, to date, there is a paucity of interventions that specifically target the satisfaction of psychological needs for these students (Malboeuf-Hurtubise et al., 2017c).

Among other potential interventions that have been proposed, the intervention *Mission Méditation*, which targets mindfulness, has shown promise (Malboeuf-Hurtubise and Lacourse, 2016). Mindfulness can be defined as the process by which we “[pay] attention in a particular way: on purpose, in the present moment, and non-judgmentally” (Kabat-Zinn, 1994). According to Ryan and Deci (2000), mindfulness could lead to optimal self-regulation by allowing individuals to be in touch with their basic psychological needs for competence (feeling effective in our actions with the environment), autonomy (feeling volition, willingness, and choice in our actions), and relatedness (feeling connected and loved by others (Ryan and Deci, 2000; Brown and Ryan, 2003). Specifically, Brown and Ryan (2003) have argued that mindfulness acts through an unveiling of moment-to-moment experiences, in which barriers such as judgmental thoughts and environmental factors clouding one’s judgment are removed. Such barriers are thought to have an impact on one’s perception of basic psychological need satisfaction. As such, mindfulness is hypothesized to lead to more realistic appraisals of the three basic psychological needs for autonomy, competence, and relatedness (Malboeuf-Hurtubise et al., 2017c), which in turn leads people to either benefit from high levels of need satisfaction or help them make the appropriate changes to improve need satisfaction. An increasing amount of research within the self-determination theory (SDT) framework has supported this proposition (Brown and Kasser, 2005; Brown et al., 2007; Levesque and Brown, 2007).

Self-determination theory is a macrotheory of human motivation and functioning that posits that the satisfaction of the three basic psychological needs are considered to be good indicators of one’s well-being (Ryan and Deci, 2000). Research suggests that in children, high satisfaction of the basic psychological needs is associated with thriving in their everyday environment (at home, in school), whereas frustration of these needs can lead to mental health issues (van der Kaap-Deeder et al., 2017). For example, in a school-based setting, a child may feel an obligation to think in a given way and thus feel that his/her autonomy need is frustrated or fail to report meaningful relationships, thus feeling his/her affiliation is frustrated. A child may also report feeling like a failure, thus feeling his/her competence is frustrated, which is a common occurrence in children with severe LDs (Malboeuf-Hurtubise et al., 2017b, c). In addition, children with severe LDs are likely to experience lower levels of need satisfaction either because of a highly controlling environment, because they find it difficult to make friends and tend to be ostracized or because they have less opportunities to experience successes at school as a direct consequence of their severe LDs (Grolnick and Ryan, 1990; van der Kaap-Deeder et al., 2017). In school settings, previous research has reported that high satisfaction of the competence need was negatively associated to depression, which is a commonly observed comorbidity to severe LDs (Véronneau et al., 2005; Bauminger and Kimhi-Kind, 2008). As such, interventions that aim to decrease symptoms of psychological disorders, while also aiming at increasing basic need satisfaction, are of special relevance for children with severe LDs. Mindfulness-based interventions (MBIs) may represent such an alternative.

Few published studies have attempted to experimentally manipulate basic psychological need satisfaction, especially in children. The available literature on interventions to foster need satisfaction targets physical education or overall sports practice, and mainly aims to increase motivation. Examples from the literature include a study by Tessier et al. (2010), in which physical education teachers were offered training to promote autonomy-supportive behaviors and to support student motivation. Results from their study showed that, following this training, teachers used more need-supportive behaviors and displayed a change in their interpersonal teaching style. Furthermore, their students also reported increases in the basic psychological need of relatedness, although autonomy and competence remained unchanged. Similar results have also been reported elsewhere, where teacher autonomy-supportive training had an impact on students’ perception of basic psychological need satisfaction, while decreasing their perception of need frustration, when compared to no-treatment controls (Cheon et al., 2016). Noteworthy, to date, no article including a control condition seems to have been published detailing an intervention tailored to increase basic psychological need satisfaction while also improving mental health in children. One quasi-experimental study presenting the impact of a parenting program has reported significant increases in autonomy support and affiliation, along with increases in well-being and overall mental health in children (Joussemet et al., 2014).

To our knowledge, only one quasi-experimental study evaluating the impact of a MBI on basic psychological needs satisfaction in children has been published to date. Results from this study showed that, although the MBI had a positive impact in reducing internalized and externalized symptoms in students with severe LDs, it was negatively related to need satisfaction (Malboeuf-Hurtubise et al., 2017b, c). These mixed findings suggest that the MBI may result in a more accurate perception of need satisfaction in these students and that less defensive appraisals could in turn improve mental health, despite students’ experiences of need insatisfaction. Given the potential of MBIs to improve the mental health of students with severe LDs and the positive impacts of MBIs on need satisfaction typically found with the adult population (Levesque and Brown, 2007), it appears crucial to further investigate the link between MBIs and need satisfaction.

Recently, MBIs have been increasingly implemented in school settings to foster better mental health and higher resilience to stress, anxiety, and depression. Results from recent meta-analyses tend to support the fact that MBIs constitute a promising option to help reduce internalized symptoms such as anxiety and depression in elementary school children, in both regular and specialized education (Zenner et al., 2014; Zoogman et al., 2014; Crescentini et al., 2016; Carsley et al., 2018). However, it appears crucial to compare MBIs to other types of interventions aimed at improving mental health (e.g., cognitive-behavioral therapy, social skills curriculum), as most studies have only used wait-list control groups, and this would allow us to eliminate potential expectancy effects as explanations for the results. Furthermore, as MBIs are costly, namely, because of its lengthy certification process, its cost-effectiveness should be further evaluated and compared to other types of interventions.

## Present Study

Malboeuf-Hurtubise et al.’s (2017c) previous study was limited by its design, as it implemented a quasi-experimental study with no control group. The present study was thus conducted in order to improve the methodological rigor of our research team’s previous pilot study (Malboeuf-Hurtubise et al., 2017c). As such, in this study, an active control condition in the form of a social skills curriculum was used, and participants were randomly allocated to either condition. Furthermore, the impact of the MBI on basic psychological need satisfaction was evaluated in conjunction with its impact on internalized symptoms, in order to grasp a more complete portrait of the MBI’s impact on mental health. Indeed, in the context of this study, mental health was evaluated with both positive (basic need satisfaction) and negative (internalized symptoms) indicators. The goal of the present study was thus to evaluate the impact of a MBI on the satisfaction of the basic psychological needs of autonomy, competence and relatedness and on internalized symptoms in students with severe LDs in a special education curriculum. In order to do so, a randomized cluster trial with an active intervention control group was implemented to document and compare the progression of basic psychological need satisfaction and symptoms of anxiety and depression in participants pre- to post-intervention and at follow-up.

### Hypotheses

Given the contradiction between our own previous preliminary findings in which a MBI had a negative impact on need satisfaction and findings from the adult literature in which mindfulness typically has a positive impact on need satisfaction (Brown and Ryan, 2003; Brown et al., 2007; Levesque and Brown, 2007), we could not formulate a definite hypothesis for the impact of the MBI on need satisfaction. Rather, we sought out to re-evaluate the impact of a MBI on need satisfaction in children with severe LDs, with an experimental longitudinal design and an active control condition.

Based on the previous literature on the impact of MBIs for children’s mental health (e.g., Zenner et al., 2014; Zoogman et al., 2014; Carsley et al., 2018), and more specifically on the impact of MBIs internalized symptoms in children with special education needs (Malboeuf-Hurtubise et al., 2017b), our first hypothesis was that the MBI would have a positive impact on children’s internalized symptoms. Our second hypothesis was that the MBI would have a significantly larger impact on mental health than the social skills curriculum used in the active control condition.

## Materials and Methods

### Participants

Twenty-three elementary school students aged 9–12 years old with severe LDs in two special education classrooms took part in this study. The two classrooms were randomly attributed to either an experimental (*n* = 13) or an active control group (*n* = 10). Students in both classrooms matched the following criteria: (a) they all had severe LDs and displayed significant academic delays of 2 years or more in reading, writing, and mathematics; and (b) they had borderline intellectual functioning (70 < IQ score > 79), as was shown by previous cognitive assessment. In order to take part in this project, participants had to consent to participate in an 8-week MBI or social skills curriculum and be available to fill out pre, post, and follow-up questionnaires. All had to have sufficient knowledge of French, as the interventions and questionnaires were provided in this language. There was no attrition in this study.

### Procedure

An experimental longitudinal randomized cluster trial with an active control condition and three assessment time points was used in this project. Participants in the experimental group completed a MBI, whereas participants in the active control group completed a social skills development curriculum. The modalities of both interventions were identical: both were delivered once a week, during 8 weeks, in a group format (i.e., in the classroom), by the same individual. There were no mindfulness activities in the social skills curriculum.

Measures were obtained at pre-test, post-test, and during a 3-month follow-up. This project was conducted in collaboration with a school board and elementary school from Vaudreuil-Dorion, Canada. Ethics approval was obtained from all institutions involved. Informed consent was obtained from all students involved in the project, their parents, and their teachers.

### Mindfulness-Based Intervention

Participants in the experimental group took part in an 8-week MBI, called *Mission Méditation*, specifically adapted and tailored to fit elementary school children’s developmental needs and attention span (Malboeuf-Hurtubise and Lacourse, 2016). This intervention was developed after many years of research in school-based settings and has demonstrated its effectiveness and appropriateness for elementary school students in regular and special education classrooms with diverse disabilities, including severe LDs, ADHD, anxiety, and depressive disorders (Malboeuf-Hurtubise et al., 2017a,b,d, 2018). The MBI is composed of eight 45- to 60-min sessions in which various meditations were introduced to students (e.g., sitting meditation, mindful stopping, mindful listening, walking meditation, and body scan). Detailed descriptions of the intervention have been published elsewhere (e.g., Malboeuf-Hurtubise et al., 2018). MBI sessions were led by a trained community involvement school counselor with prior experience and extensive training in mindfulness practice. Sessions were scheduled to occur once a week, and the MBI condition teacher was asked to practice concepts at least once in between sessions. The intervention did not include a silent retreat.

### Social Skills Curriculum

Participants in the active control group took part in an 8-week social skills curriculum, provided by the same community involvement school counselor. This curriculum was an in-house school-board-wide program in which the students were exposed to different social skills activities, with the overarching goal of finding purpose in life, becoming responsible and engaged citizens, and developing a sense of belonging to the school and community (Commission Scolaire des Trois-Lacs, 2015). The following themes were explored as part of this intervention: using your own personal strengths, using classmates’ personal strengths, finding meaning and pleasure in life, and being creative.

### Measures

Both measures chosen for this study have been shown as valid in a sample of children with severe LDs (Malboeuf-Hurtubise et al., 2017b, d).

Participants rated how competent, autonomous, and related they felt in school, by answering a nine-item scale adapted from a scale used in a previous, similar study (Savard et al., 2013; Malboeuf-Hurtubise et al., 2017c). Children were asked to rate their agreement to items such as “In school, I feel free to be myself’(autonomy); I am able to reach my goals” (competence) and “In my relationship with others, I feel appreciated” (relatedness). Internal consistency was acceptable (α = 0.74).

Participants also completed selected items from the anxiety (three items, e.g., “I worry about little things”) and depression (five items, e.g., “Nothing ever goes right for me”) subscales of the *Behavior Assessment Scale for Children* (BASC II) (Reynolds and Kamphaus, 2004). Internal consistency was acceptable for both subscales (α _depression_ = 0.61; α _anxiety_ = 0.76).

### Data Analysis

Hypotheses were tested using mixed ANOVAs allowing comparison of pre-to-post and follow-up scores as moderated by the experimental condition. Effect sizes were also computed in order to assess the magnitude of the observed effects.

### Statistical Power

An *a priori* statistical power calculation was done using G^∗^Power software (Faul et al., 2007) and showed that, in order to ensure a statistical power of 0.8, with three assessment time points, two groups, and a moderate effect size (*F* = 0.25), a total sample size of 28 students was required to ensure sufficient statistical power in this study. *A posteriori* statistical power analyses were conducted using the following parameters: effect size *F* (converted η^2^ value), α error probability (0.05), total sample size (23 participants), number of groups (2), number of measurements (3), correlation among measures for each variable (values can be found in Tables 2 and 3), and non-sphericity correction ∈ (obtained from Mauchly’s sphericity test in SPSS = 1 for each variable). Power associated with each analysis can be found in Tables 2 and 3.

## Results

Preliminary analyses using independent *t-*tests first showed that the two groups differed at pre-intervention [*t*(21) = −2.80, *p* = 0.01] only on measures of competence. Mixed ANOVAs revealed that these initial differences were unaffected by the experimental manipulation, suggesting that the effects in the experimental and active control groups were similar over time for all dependent variables. Detailed results can be found below and in Tables 1–3.

**TABLE 1:** Means and standard deviations for basic psychological need satisfaction, anxiety and depression symptoms.

	**Control group**			**Experimental group**		
**Dependent variable**	**Pre-test (SD)**	**Post-test (SD)**	**Follow-up (SD)**	**Pre-test (SD)**	**Post-test (SD)**	**Follow-up (SD)**
**Need satisfaction**						
Total need satisfaction	8,58(2,69)	8,96(2,31)	9,74(2,47)	10,26(1,30)	9,60(2,16)	10,26(2,25)
Autonomy	8,92(2,60)	8,12(3,21)	9,66(3,97)	9,67(1,43)	8,85(2,38)	10,23(2,46)
Competence	8,44(3,20)	9,36(2,99)	11,19(2,03)	11,39(1,83)	12,28(2,26)	11,81(1,10)
Relatedness	8,38(3,56)	9,38(4,05)	7,92(2,50)	9,70(2,00)	7,69(3,25)	8,75(3,38)
**Mental health**						
Anxiety	3,50(2,72)	1,99(2,89)	1,22(4,37)	3,83(2,34)	2,43(1,50)	2,78(1,65)
Depression	6,51(4,95)	2,09(8,14)	1,83(7,16)	4,12(3,72)	3,14(3,10)	4,18(4,07)

**TABLE 2:** Results of Mixed ANOVAs for basic psychological need satisfaction.

	**Total need satisfaction**					**Autonomy**					**Competence**					**Relatedness**				
	**Overall model**					**Overall model**					**Overall model**					**Overall model**				
	**df**	***F***	***p***	**Partial η^2^**	**Power**	**df**	***F***	***p***	**Partial η^2^**	**Power**	**df**	***F***	***p***	**Partial η^2^**	**Power**	**df**	***F***	***p***	**Partial η^2^**	**Power**
Time	2,20	0.99	0.39	0.09	0.89	2,20	1.7	0.21	0.15	0.99	**2,20**	**5.10**	**0.02^∗^**	**0.34**	**1.00**	2,20	0.40	0.67	0.04	0.52
Group condition	1,21	1.91	0.18	0.08	0.85	1,21	0.79	0.39	0.04	0.52	**1,21**	**7.03**	**0.02^∗^**	**0.25**	**0.99**	1,21	0.03	0.86	0.001	0.06
Time × Group condition	2,20	0.61	0.56	0.06	0.72	2,20	0.01	0.99	0.001	0.06	2,20	1.99	0.16	0.17	0.99	2,20	2.45	0.11	0.20	0.99

*^∗^*p* ≤ 0.05, ^∗∗^*p* ≤ 0.01. Bolded values are simply those that are statistically significant.*

**TABLE 3:** Results of mixed ANOVAs for anxiety and depression symptoms.

	**Anxiety**					**Depression**				
	**Overall model**					**Overall model**				
	**df**	***F***	***p***	**Partial η^2^**	**Power**	**df**	***F***	***p***	**Partial η^2^**	**Power**
Time	**2,20**	**6.19**	**0.008^∗∗^**	**0.38**	**1.00**	2,20	1.35	0.28	0.12	0.96
Group condition	1,21	0.35	0.56	0.02	0.28	1,21	0.40	0.84	0.002	0.07
Time × Group condition	2,20	0.26	0.78	0.03	0.39	2,20	2.50	0.11	0.20	0.99

*^∗^*p* ≤ 0.05, ^∗∗^*p* ≤ 0.01. Bolded values are simply those that are statistically significant.*

### Basic Psychological Need Satisfaction

The experimental condition thus did not moderate change in participants’ scores of autonomy, competence, and relatedness across the three time periods. Looking at main effects of time, we found no within-person difference on scores of autonomy and relatedness (please refer to Tables 1 and 2). However, a significant main effect was found for the competence need over time [Wilks Lambda = 0.66, *F*(2,20) = 5.10, *p* = 0.02, partial η^2^ = 0.34]. Bonferroni-adjusted *post hoc* analyses showed that for both groups, scores were significantly higher at post-intervention (*p* = 0.03) and at follow-up (*p* = 0.04), when compared to pre-intervention scores. There was no significant difference between scores at post-intervention and follow-up (*p* = 0.76). Hence, participants from both groups displayed higher competence scores from pre- to post-intervention, and these improvements remained stable at follow-up.

### Internalized Symptoms

The experimental condition also did not moderate change in participants’ scores of depression and anxiety across the three time periods. Looking at main effects of time, we found no within-person difference on scores of depression (please refer to Tables 1 and 3). However, a significant main effect was found for anxiety levels over time [Wilks Lambda = 0.61, *F*(2,42) = 6.19, *p* = 0.008, partial η^2^ = 0.38]. Bonferroni-adjusted *post hoc* analyses showed that for both groups, scores were significantly lower at post-intervention (*p* = 0.01) and at follow-up (*p* = 0.006), when compared to pre-intervention scores. There was no significant difference between scores at post-intervention and follow-up (*p* = 0.49).

## Discussion

Results from this study show that although the MBI appears to be useful in increasing the basic psychological need of competence and decreasing anxiety in students with severe LDs, it was not more useful than the active control intervention that was used in this project. These findings contradict previous results in which a MBI led to decreases in need satisfaction of students with severe LDs (Malboeuf-Hurtubise et al., 2017c). These seemingly more intuitive results are in line with those reported in adults in the literature on SDT and mindfulness (Brown and Ryan, 2003; Brown et al., 2007; Levesque and Brown, 2007). However, although this study was more rigorous in its attempt to implement an experimental design with an active control group, this rigor alone cannot explain these opposite results, as one would not expect the addition of a control group to change students’ perception of need satisfaction.

One potential explanation for these results may lie in the need satisfaction scale that was used in this project and in our previous study. This scale measures how children’s basic needs are satisfied when they are at school, which means that it is highly context-dependent. Since mindfulness training allows children to “see more clearly,” to become aware of their environment for what it is, a child who has become more mindful and who evolves in a classroom where the teacher is need-supportive will become more aware of this and might have a higher need satisfaction score after the intervention. On the other hand, a child who has become more mindful and more aware of a less need-supportive, or even controlling, classroom context may rate their need satisfaction as lower than at the beginning of the intervention. It has been proposed that children’s perceptions are likely to be a direct consequence of environmental feedback rather than the product of internal factors (Abela and Taylor, 2003), especially given that they have not reached the formal operational stage of cognitive development. In order to disentangle this, future studies should compare elementary school children to adolescents whose perceptions are less likely to be a direct product of the school context. Another option would also be to evaluate directly and control for classroom climate in future studies. Moreover, these studies should use a broader measure of need satisfaction to capture the different environments that the children are exposed to in their life.

For now, we observe that the present results are in line with recent findings and meta-analyses published on the impact of MBIs for youth in school-based settings, in clinical and non-clinical populations (Zenner et al., 2014; Zoogman et al., 2014; Carsley et al., 2018). However, in this sample, the MBI was not more useful to increase basic psychological need satisfaction or to improve mental health than the active control intervention, in which a social skills curriculum was offered. Others studies comparing the impact of MBIs to active control conditions have reported similar positive – yet not significantly different from the control intervention – effects, namely, in elementary school students from regular classrooms (Britton et al., 2014) and in a variety of other contexts [e.g., teenagers with eating disorders (Atkinson and Wade, 2015); mindful coloring in elementary school students (Heath and Fajnerova, 2015)]. In a recent meta-analysis published by Carsley et al. (2018), the authors were not able to perform impact analyses with regards to the type of control condition, given the small number of studies in which MBIs were compared to active control conditions. However, the available evidence suggests that although MBIs can be useful to improve mental health and well-being in children, perhaps they are not the most effective, or cost-effective, interventions. Indeed, as mindfulness certification is a long process requiring several months or even years of personal practice and training, it appears relevant to question its added value in school-based settings. Furthermore, given previous results in which a MBI had a deleterious impact on need satisfaction in youth with severe LDs, this ongoing reflection should warrant serious attention and thought from the research community (Malboeuf-Hurtubise et al., 2017c; Baer et al., 2019).

### Strengths and Limitations

This study counts notable strengths, among which the most important resides in its design. Indeed, this study used a randomized cluster trial with an active control condition. Studies evaluating the impact of MBIs in children have historically lacked rigor in their designs, and, as such, we have aimed to correct for this (Carsley et al., 2018). Furthermore, this study is one of few that aims to document the impact of MBIs on basic psychological needs satisfaction, specifically for students with severe LDs, whereas the vast majority of the published literature on this subject is correlational. Another strength of the present study is that there was no attrition such that our main findings could not be explained by differences in the sample across time points.

Despite these strengths, the small sample size represents an important limitation of this project. Indeed, a larger randomized cluster trial would have provided more robust and generalizable results, and should thus be planned as future steps in this line of research. Increasing the sample size would also help to ensure meeting minimal requirements for adequate statistical power. As such, given this final sample size fell short of a few participants to meet such requirements, it is possible that we were not able to detect significant pre-to-post changes in participant scores and across conditions for some variables, increasing the risk of making a Type II error. Choosing different measures for future research studies may also be recommended, as the internal consistency for both scales in this project was acceptable, but could have shown higher values. Administering a more important number of items could help solve this issue, as only a few selected items were administered in the current project, thus reducing the sensitivity of our measures to changes in our participants. It could thus be possible to detect significant pre-to-post changes with a larger number of administered items. We note, however, that with this sample size and this number of items, we were able to detect changes in perceived competence and anxiety scores across time points. Finally, adding specific items measuring need frustration could also have been helpful in getting a broader and more complete picture of how mindfulness can have an impact on basic psychological needs, although existing scales would require an adaptation to be adequately understood by children (Chen et al., 2015). Finally, given that improvements were observed in participants from both conditions, it remains possible that the documented effects were not due to the interventions themselves, but rather to the simple passage of time.

### Suggestions for Future Studies

Future research is needed to determine the impact of MBIs on need satisfaction for students with special education needs. Optimal contexts for implementation would also need to be determined, as the removal of barriers clouding one’s appraisals of basic psychological need fulfillment may not always be optimal for children. Indeed, compared to adults, children have less control over their situation in school or at home such that making the changes needed to increase their satisfaction of autonomy, competence, and relatedness when these are found to be low may not always be possible. One way to correct this issue could be to train teachers themselves to become generally more mindful and specifically more mindful of their actions and words toward their students, as previous research has shown that teachers in special education classrooms tend to be more controlling and less autonomy supportive than their colleagues in regular classrooms (Grolnick and Ryan, 1990). It has been suggested that teachers can be trained in mindfulness using a program called CARE (Cultivating Awareness and Resilience in Education), to become more supportive of their students and promote their learning and engagement (Taylor, 2017). In fact, a recent randomized control trial among 224 teachers showed that, compared with control teachers, CARE teachers felt less stressed and more mindful and provided more emotional support and structure to students as observed by independent raters (Jennings et al., 2017). This research shows that a MBI specifically designed for teachers improves their functioning and influences their behavior in class to be more supportive of their students. Future studies should evaluate whether increasing teachers’ own levels of mindfulness influences how much they support their students’ autonomy, competence, and relatedness, according to SDT.

Although teachers seem to be familiar with concepts of self-determination and basic psychological need satisfaction, and consider them to be important in the classroom, the extent to which they use specific autonomy-supportive practices (e.g., self-instruction, opportunities to make choices in classroom routine, self-scheduling, and goal setting) varies greatly and is not reflective of the importance given to these concepts (Wehmeyer et al., 2000). This translation issue from theory to practice seems even more problematic for teachers in special education classrooms who work with children with severe LDs and, as such, warrants close attention and guidance from the research and clinical community.

It is also suggested that future studies investigate the possible interplay between mindfulness and sensory processing patterns in conditions such as severe LDs (Serafini et al., 2017). Past studies have highlighted the involvement of sensory perception in emotional processes, with, namely, depressed and anxious adults displaying a general hyposensitivity profile (Engel-Yeger et al., 2016). As such, sensory processing patterns might be “trait” markers of individuals with neuropsychiatric conditions and psychological disturbances. Importantly, the involvement of extreme sensory processing patterns has been hypothesized to contribute to the complex pathophysiology of these conditions. The careful assessment of sensory profiles and of their possible interaction with mindfulness may help in developing targeted interventions and improve functional/adaptive strategies in children with severe LDs.

Finally, in light of our results, future studies should verify that MBIs have an added value compared to other types of interventions, especially in cases where these other interventions are more easily implemented in school-based settings (e.g., requiring less intensive training for school professionals or teachers). Further research comparing the effectiveness of MBIs to other types of school-based interventions is thus warranted.

## Ethics Statement

This study was carried out in accordance with requirements of the Research and Ethics Committee of the Université du Québec à Montréal and Université du Québec en Outaouais, in Montreal and Gatineau, Canada. The protocol and study were reviewed and approved by both institutional Research and Ethics Committees, responsible for granting ethics approvals. All subjects and their parents gave written informed consent in accordance with the Declaration of Helsinki.

## Author Contributions

CM-H and GT conceptualized and coordinated the study, adapted the mindfulness-based intervention and trained the school counselor involved in this study, performed data analysis, and drafted the manuscript. GT contributed extensively to data interpretation and revision of the manuscript. GM contributed significantly in data analysis and to the revision of the manuscript.

## Conflict of Interest

The authors declare that the research was conducted in the absence of any commercial or financial relationships that could be construed as a potential conflict of interest.
